# Pseudocin 196, a novel lantibiotic produced by *Bifidobacterium pseudocatenulatum* elicits antimicrobial activity against clinically relevant pathogens

**DOI:** 10.1080/19490976.2024.2387139

**Published:** 2024-08-06

**Authors:** Rocio Sanchez-Gallardo, Paula M. O’Connor, Ian J. O’Neill, Brian McDonnell, Ciaran Lee, Rebecca L. Moore, Fionnuala M. McAuliffe, Paul D. Cotter, Douwe van Sinderen

**Affiliations:** a APC Microbiome Ireland, University College Cork, Cork, Ireland; b School of Microbiology, University College Cork, Cork, Ireland; c Food Biosciences, Teagasc Food Research Centre Moorepark, Cork, Ireland; d UCD Perinatal Research Centre, School of Medicine, University College Dublin, National Maternity Hospital, Dublin, Ireland

**Keywords:** Bifidobacteria, bacteriocin, antimicrobial peptide, gut microbiota

## Abstract

Bacteriocins are broad or narrow-spectrum antimicrobial compounds that have received significant scientific attention due to their potential to treat infections caused by antibiotic-resistant pathogenic bacteria. The genome of *Bifidobacterium pseudocatenulatum* MM0196, an antimicrobial-producing, fecal isolate from a healthy pregnant woman, was shown to contain a gene cluster predicted to encode Pseudocin 196, a novel lantibiotic, in addition to proteins involved in its processing, transport and immunity. Following antimicrobial assessment against various indicator strains, protease-sensitive Pseudocin 196 was purified to homogeneity from cell-free supernatant. MALDI TOF mass spectrometry confirmed that the purified antimicrobial compound corresponds to a molecular mass of 2679 Da, which is consistent with that deduced from its genetic origin. Pseudocin 196 is classified as a lantibiotic based on its similarity to lacticin 481, a lanthionine ring-containing lantibiotic produced by *Lactococcus lactis*. Pseudocin 196, the first reported bacteriocin produced by a *B. pseudocatenulatum* species of human origin, was shown to inhibit clinically relevant pathogens, such as *Clostridium* spp. and *Streptococcus* spp. thereby highlighting the potential application of this strain as a probiotic to treat and prevent bacterial infections.

## Background

Bacteriocins are ribosomally synthesized antimicrobial peptides, which display narrow (targeting closely related species only) or broad (also targeting unrelated species)-spectrum activity against bacteria, including drug-resistant strains, their mechanism of action being distinct from those elicited by pharmaceutical antibiotics.^1^ Similar to antibiotics, resistance or insensitivity to bacteriocin action has been reported.^2,3^ Bacteriocins produced by Gram-positive bacteria are generally divided into two classes namely, Class I, represented by ribosomally synthesized post-translationally modified peptides (RiPPS), and Class II, members of which typically represent unmodified peptides.^1,4^ Class I bacteriocins include lantibiotics, which are small peptides that undergo post-translational modifications whereby serines, threonines and cysteines are dehydrated and cyclized resulting in the unusual amino acids lanthionine and methyl-lanthionine, that form their characteristic internal ring structures. These thioether ring structures are essential for activity and confer significant heat, pH, and protease resistance.^5^ Lantibiotics are encoded as prepeptides by structural genes (*lanA*) which are found on gene clusters together with genes involved in their modification (*lanB*, *lanC*, *lanM*), processing (*lanP*), transport (*lanT*) and immunity (*lanI*, *lanFEG*), and exert their antimicrobial effect through pore formation and/or inhibition of cell wall biosynthesis leading to death of the target cell.^5,6^ Interestingly, lantibiotics can be further divided into type I, where lanthionines are formed by two separate enzymes (i.e., a dehydrase and cyclase encoded by *lanB* and *lanC*, respectively), and type II where these reactions are carried out by a single LanM enzyme encoded by *lanM*.^6^

Bacteriocins, initially developed as natural food preservatives, are now being investigated for veterinary and clinical use as antimicrobial, antiviral and anticancer agents. They are of interest as antibiotic alternatives due to their inherent stability, ability to kill at low concentrations and target specificity.^7^ In addition, bacteriocin-producing probiotic strains are considered microbiome modulators that can improve gut health through pathogen inhibition, immune modulation and inflammation prevention.^7,8^ Species belonging to the genus *Bifidobacterium* represent common human gut commensals, with some reported to encode bacteriocins,^9–12^ although very few bifidobacterial strains are known to actually produce antimicrobial peptides.^13,14^ One of the first bacteriocins reported in a *Bifidobacterium* species was Bifidocin B, a class II bacteriocin whose production, though not its immunity, was presumed to be plasmid associated.^15^ Lee and coworkers reported in 2008 that *Bifidobacterium longum* strain DJO10A possesses a bacteriocin cluster and was producing a lantibiotic when grown on agar but not in broth. Subsequent transcriptional analysis of that gene cluster revealed that these genes were highly regulated during log phase, describing for the first time transcriptional analysis of a two-component-regulated, bifidobacterial promoter.^13,16^ In 2010, Cheikhyoussef and colleagues described the purification of Bifidin I, anon-lantibiotic antimicrobial peptide produced by *Bifidobacterium longum* subsp. *infantis* BCRC14602. Bifidin I is a broad spectrum bacteriocin whose production, like that of Bifidocin B, is associated with the presence of a plasmid. Recently, *B. longum* subsp. infantis LH_664 strain was reported to contain genes necessary for Bifidococcin_664 biosynthesis, though production of an active peptide could not be confirmed. To overcome this the peptide was chemically synthesized and characterized.^17,18^ These studies which span more than 20 years of research highlight the apparent scarcity of bacteriocin-production among bifidobacterial strains.

Bifidobacteria typically inhabit the gastrointestinal tract (GIT) of humans, other mammals and birds, and various probiotic or health-promoting traits have been attributed to particular bifidobacterial strains.^19–21^ Being prevalent and abundant colonizers of the neonatal gut, bifidobacteria play a key role in the development and maturation of the infant gut. Indeed, a bifidobacteria-rich gut microbiota is considered to support infant health and well-being.^20,22^ A recent study attributed some of these benefits to the production of aromatic amino acid-derived metabolites, in particular indole lactic acid, which is involved in the fortification of the intestinal barrier and protection against pathogenic infections.^23^

Neonatal infections are especially common in premature babies, being frequently caused by opportunistic pathogens, against which the immature immune system has little or no defense. Necrotizing enterocolitis (NEC) is a very serious consequence of such an infection, being the most common cause of gastrointestinal morbidity and mortality in premature infants.^24^ NEC is a severe illness which manifests itself as an acute inflammation of the neonatal intestine, and which typically occurs within the first two weeks following birth, especially among preterm babies. *Clostridium* spp. such as *C. perfringens* and *C. butyricum*, as well as *Clostridioides difficile* among others are involved in the pathogenesis of NEC, corroborated by the observation that infants colonized with certain *Clostridium* species show a rapid progression of the disease.^25,26^ This is in particular the case for *C. perfringens* colonization, as this species may produce α-toxin causing a fulminant form of NEC, which is associated with higher morbidity and mortality rates compared to non-*C. perfringens* NEC cases.^27,28^ The incidence of NEC among newborns is 8.8%, according to a recent study carried out in Spain.^29^ This disease has a mortality rate of 35% in extremely low birth weight infant and survivors may suffer from gastrointestinal dysfunction that can last their lifetime.^30^

Another common neonatal infection is caused by *Streptococcus agalactiae*, which is a Group B *Streptococcus* (GBS) species, and which is the main causative agent of invasive bacterial infection, being the leading cause of postpartum infection and neonatal sepsis.^31,32^ This infection causes adverse maternal and newborn outcomes, annually causing nearly half a million infections and over 100,000 stillbirths and infant deaths.^33^

In the current study, we describe the full genome sequence of *Bifidobacterium pseudocatenulatum* MM0196, a fecal isolate originating from a healthy pregnant woman displaying antimicrobial activity against a number of Gram-positive indicator strains. Analysis of more than 400 genome sequences of bifidobacteria isolated from mother-infant dyads, revealed that several harbor gene clusters predicted to be involved in bacteriocin production though only one strain displayed antimicrobial activity under the conditions tested. *In silico* analysis of its genome revealed a novel gene cluster predicted to be involved in the production of a lantibiotic hereby named pseudocin 196. Purification of the antimicrobial compound from cell-free supernatant (CFS) to homogeneity confirmed production of pseudocin 196 a lantibiotic active against clinically-relevant bacteria such as various pathogens belonging to the genera *Streptococcus* and *Clostridium*.

## Results

### *Isolation and whole genome sequencing of* B. pseudocatenulatum *MM0196*

The characterization of human derived *Bifidobacterium* strains with a view to explore their possible probiotic properties, and antimicrobial activities in particular, was the focus of the current study. Over 500 bifidobacterial strains had previously been isolated and sequenced as part of the “Microbe Mom” project, a clinical trial aimed at assessing vertical transfer of bifidobacterial strains between mother-infant dyads (TRN: ISRCTN53023014).^34^ The obtained bifidobacterial genome sequences were scrutinized by *in silico* analysis employing BAGEL4^35^ in order to detect (novel) bacteriocin gene clusters. Of the 501 bifidobacterial genomes analyzed, 64 harbored genes typical of those involved in bacteriocin production.

**Figure 1.**
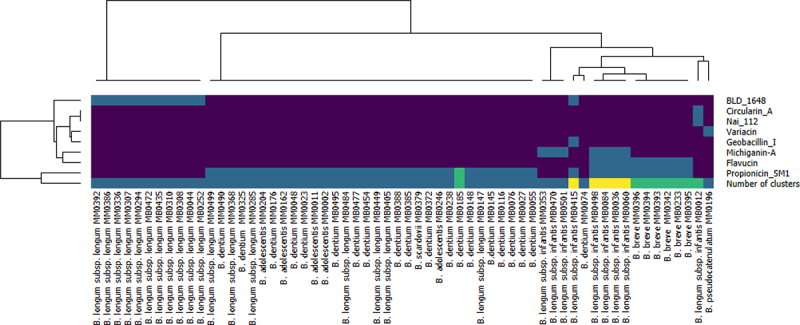
BAGEL4 prediction. Heatmap represents the presence/absence of bacteriocin-like cluster in the strains where at least one cluster was predicted (dark blue means the cluster is absent, light blue, green or yellow means the cluster(s) is (are) present). The last row of the heatmap represents the total number of clusters predicted in each genome (dark blue = 0, light blue = 1, green = 2 and yellow = 3).

Eight distinct bacteriocin production clusters were predicted based on their similarity to known bacteriocin gene clusters: BLD_1648, Propionicin_SM1, Flavucin, Michiganin-A, Circularin_A, Nai_112, Variacin and Geobacillin_I_like (Supplementary material: Figure S1). In some cases, up to three clusters were predicted in a single genome, i.e., for strains MB0036, MB0060, MB0084, MB0415, MB0498, and MB0501. Interestingly, all these strains belong to the species *B. longum* subsp. *infantis* (Figure 1). In order to assess if strains carrying such predicted bacteriocin clusters actually produce an antimicrobial peptide these bifidobacterial strains were screened employing the so-called agar-overlay approach and well diffusion assay (see Materials and Methods). At least one strain containing each distinct cluster, or combination of clusters was tested. A total of 55 strains were tested, however, just one, *B. pseudocatenulatum* MM0196, was shown to exhibit clear antimicrobial activity when its cell free supernatant was tested against a variety of indicator strains by well diffusion assay (supplementary data). This strain and the antimicrobial it produces were therefore selected for further characterization.

### *In silico analysis of the* B. pseudocatenulatum *genome reveals a bacteriocin-encoding gene cluster*

**Figure 2.**
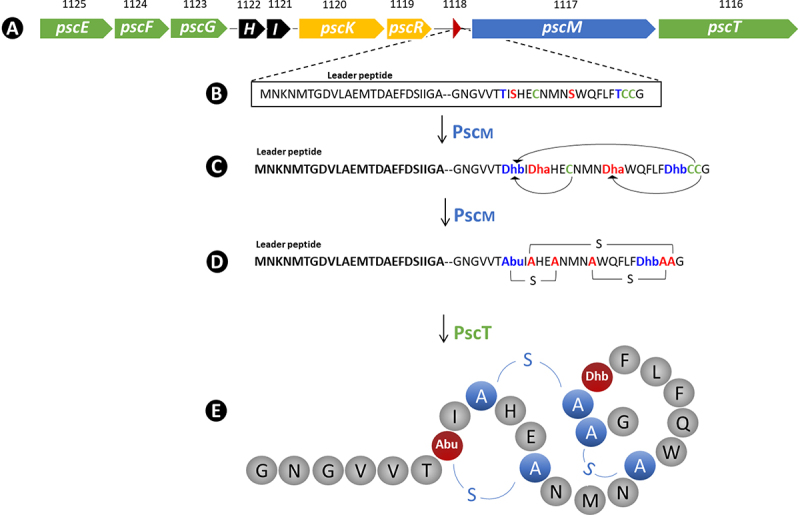
Schematic representation of the bacteriocin-encoding *psc* gene cluster as revealed by genome sequencing (A). The amino acid sequence of the unmodified pro-peptide is shown for the structural gene *pscA* (B). The product of *pscM* is predicted to dehydrate serines and threonines to dehydroalanine (dha) and dehydrobutyrine (dhb), respectively (C), and forms lanthionine (represented as A-S-A) and methyl lanthionine rings (represented as abu-S-A in order to keep consistency with previous studies^37,38^) (D), while PscT is predicted to remove the signal peptide during transportation across the cell membrane resulting in active pseudocin 196 (E).^38^

As mentioned above, the *in silico* screen for bacteriocin-encoding genes in the genome of *B. pseudocatenulatum* MM0196 identified a single 11,134 bp gene cluster predicted to be responsible for the production of a type II lantibiotic with high similarity to variacin produced by *Micrococcus varians*.^36^ The gene cluster, referred to here as the *psc* gene cluster, encompasses a total of 10 genes flanked on either side by an IS element (Figure 2). The genes corresponding to locus tags MM0196_0183, MM0196_0184, and MM0196_0185, designated here as *pscE*, *pscF*, and *pscG*, respectively, are predicted to encode an ABC transporter system with a putative role in bacteriocin immunity. The next two genes corresponding to locus tags MM0196_0186 and MM0196_0187, designated here as *pscH* and *pscI*, encode proteins of unknown function. The genes with locus tags MM0196_0188, MM0196_0189, designated as *pscK* and *pscR*, encode a putative histidine kinase and a response regulator, respectively, which are expected to be involved in transcriptional regulation of the bacteriocin cluster. The gene associated with locus tag MM0196_0190, designated here as *pscA*, encodes the putative bacteriocin pro-peptide, which is expected to be modified by the product of *pscM* (MM0196_0191), predicted to be a lanthipeptide synthetase. Finally, the *pscT* gene with locus tag MM0196_0192 is the last gene of the cluster and is likely to encode a transporter with a protease domain involved in the processing and concomitant export of PscM-modified PscA to produce the biologically active bacteriocin, which we henceforth refer to as pseudocin 196 (Supplementary Table S2).

**Figure 3.**
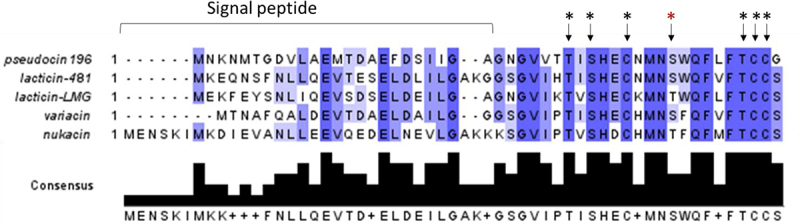
Alignment of pseudocin 196 with previously described lantibiotics produced by other Gram-positive bacterial species. Arrows indicate the amino acid residues involved in the thioether linkages (black asterisk: conserved; red asterisk: non conserved). Alignment visualised with Jalview.

The 150 bp (including stop codon) *pscA* gene encodes a 49 amino acid peptide, with the first 22 corresponding to the signal peptide (as predicted using SignalP 5.0 software), removal of which is predicted to result in the mature 27 amino acid PscA. A non-redundant blast search of the PscA primary amino acid sequence indicates that it represents a novel bacteriocin, most similar to several known lantibiotics: lacticin 481 (58% with signal peptide/80% without signal peptide) produced by particular *L*. *lactis* strains, lacticin LMG (57.14% with signal peptide/80% without signal peptide) produced by *L. lactis* LMG2081, variacin (69.23% with signal peptide/72% without signal peptide) produced by *Micrococcus varians*, and nukacin ISK-1 (41.30% with signal peptide/58.33% without signal peptide) produced by *Staphylococcus warneri* ISK-1 (Figure 3). The diversity of the pro-peptide is mostly located in the N-terminal signal peptide sequence, while the region corresponding to the pro-peptide is highly conserved. Notably, these lantibiotics are produced by Gram positive bacteria that are not closely related.

Sequence alignment of the pro-peptides from the above-mentioned lantibiotics shows that serines (Ser), threonines (Thr), and cysteines (Cys) are fully conserved, being consistent with their predicted role in lanthionine ring formation. Based on predicted ring formation in lacticin 481, it is proposed that rings are formed between Thr7 (threonine at position 7 in the mature PscA) and Cys12, Ser9 and Cys23, Ser16 and Cys24 with Thr22 being modified to dehydrobutyrate (DHB). These modifications result in a lantibiotic with two lanthionine rings, a single methyl lanthionine ring and one DHB residue, as highlighted in Figure 2(E). The calculated molecular mass of the mature, unmodified PscA peptide is 2747.16 Da, while the formation of three thioether rings and a threonine to dehydrobutyrate dehydration would result in a 72 Da molecular mass reduction, resulting in a predicted mass of the mature, fully processed lantibiotic, pseudocin 196, of 2675.16 Da.

As bacteriocin production has not previously been reported in *B*. *pseudocatenulatum*, all publicly available *B. pseudocatenulatum* genomes were analyzed by Bagel 4 to determine the frequency of putative bacteriocin-producing genes in this species. Among 103 bioinformatically assessed genomes, just two were predicted to harbor a putative bacteriocin gene cluster. The *B. pseudocatenulatum* SS Bg39 genome (accession no. GCA_902167925.1) is predicted to harbor genes responsible for the production of propionicin, while *B. pseudocatenulatum* CA-K29b (accession no. GCA_001686065.1) was predicted to encode a variacin-like gene cluster, being identical to the pseudocin 196 gene cluster of *B. pseudocatenulatum* MM0196. The genomes of *B. pseudocatenulatum* strains SS Bg39 and CA-K29b represent so-called MAGs (metagenomic assembled genomes) and isolation of these strains has not been reported.

The presence of two adjacent transposase-encoding genes flanking the pseudocin 196 gene cluster and the lower GC content of the gene cluster (54.44%) compared to the rest of the genome (56.56%) suggests that the cluster was horizontally acquired from another bacterium present in the same ecological niche. To elucidate the possible origin of the pseudocin 196 gene cluster, a phylogenetic tree was constructed based on a multiple sequence alignment with the first 100 results of a non-redundant blast using the amino acid sequence of the lantibiotic peptide (supplementary material). The generated phylogenetic tree shows that most of the bacteria encoding similar peptides are human-associated species, consistent with the common niche-mediated horizontal transference hypothesis.

### *Confirmation of pseudocin 196 production by* B. pseudocatenulatum *MM0196*

**Figure 4.**
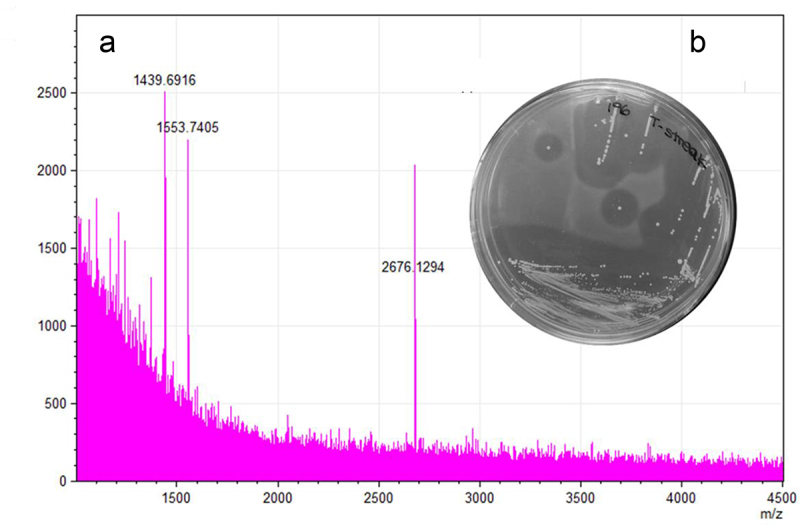
A 2676.13 Da mass detected by MALDI-TOF MS from a colony grown in RCA (reinforced clostridial agar) correlates with the theoretical mass of the modified bacteriocin (2676.13 Da) (a). Antimicrobial activity shown by overlay assay against *L. cremoris* HP (b).

To determine if *B. pseudocatenulatum* MM0196 is producing the predicted lantibiotic, the strain was tested for antimicrobial activity in an overlay assay against *Lactococcus cremori*s HP, an indicator strain selected based on its reported sensitivity to lacticin 481.^39^ The presence of a zone of inhibition verified that the strain was active against *L. cremori*s HP (Figure 4(b)), while colony MALDI TOF mass spectrometry analysis detected a molecular mass of 2676.13 Da (Figure 4(a)), which corresponds exactly to the theoretical mass of the protonated (molecular mass +1 Da from the addition of [H+]) putative pseudocin 196, this being 2676.16 Da (i.e., 2675.16 Da +1 Da [H+]).

Pseudocin 196 activity is detectable following 6 h of cultivation from initial inoculation, corresponding to mid logarithmic growth while maximum antimicrobial activity was observed at the late stationary phase, corresponding to 28 h of cultivation. Antimicrobial-containing CFS was exposed to different temperatures and pH values to assess stability of the antimicrobial activity. Pseudocin 196 was shown to be very heat stable as activity was detected against *L. cremoris* HP even after autoclaving showing that extreme temperature exposure does not cause significant loss of antimicrobial activity against *L. cremoris* HP when assessed by well diffusion assay. Similarly, exposure to high or low pH did not diminish the antimicrobial activity under the conditions tested (data not shown). The antimicrobial activity was successfully purified from CFS using hydrophobic interaction amberlite XAD16N beads, C18 Solid Phase Extraction (C18 SPE) and reversed phase HPLC resulting in a pure peptide with antimicrobial activity and a mass of 2676 Da (data not shown), concordant with the deduced molecular mass of pseudocin 196. The above findings demonstrate that the observed antimicrobial activity is due to a single, heat and pH resistant compound corresponding to pseudocin 196.

### Inhibitory spectrum of pseudocin 196

CFS of *B. pseudocatenulatum* MM0196 was used to assess the antimicrobial activity of pseudocin 196 against a range of Gram-positive bacteria using the well diffusion assay. These well diffusion assays, based on the diameter size of the inhibition zone, revealed that some of the strains tested were indeed sensitive to the antimicrobial activity present in the CFS, as summarized in Table 1, with the most sensitive strain being *L. cremori*s HP. Overall the spectrum of inhibition shows that pseudocin 196 is active against a broad range of Gram positive micro-organisms similar to those obtained for other bacteriocins belonging to the lacticin 481 group.^36,40,41^

**Table 1.** Spectrum of inhibition of cell free supernatant of *B. pseudocatenulatum* MM0196 against a range of bacterial strains.

Species	Growth conditions		Inhibition*
*Lactococcus cremoris* HP		GM17,30°C, aerobic	+++
*Lactococcus cremoris NZ9000*		GM17,30°C, aerobic	++
*Lactococcus coryniformis* subsp. *torquens* 14I		MRS,37°C, aerobic	−
*Lactobacillus salicei* 42C		MRS, 30°C, aerobic	+
*Lactobacillus curvatus* 40A		MRS, 30°C, aerobic	++
*Lactobacillus plantarum* 41P		MRS, 30°C, aerobic	−
*Listeria innocua* UCC1		BHI, 37°C, aerobic	−
*Enterococcus faecalis* 4F		BHI, 37°C, aerobic	−
*Streptococcus dysgalactiae* GpB		BHI, 37°C, aerobic	++
*Streptococcus agalactiae* ATCC 13,813		BHI, 37°C, aerobic	+
*Staphylococcus aureus* NCOO 94		BHI, 37°C, aerobic	−
*Pseudomonas aeruginosa* NCIMB 10,421		BHI, 37°C, aerobic	−
*Bifidobacterium bifidum* NCIMB 8810		MRS/RCM, 37°C, anaerobic	−
*Bifidobacterium breve* UCC2003		MRS/RCM, 37°C, anaerobic	−
*Bifidobacterium adolescentis* NCFB 2229		MRS/RCM, 37°C, anaerobic	+
*Bifidobacterium longum* NCIMB 8809		MRS/RCM, 37°C, anaerobic	+
*Bifidobacterium longum* CCUG 52,486		MRS/RCM, 37°C, anaerobic	−
*Bifidobacterium* subsp. *infantis* ATCC 15,697		MRS/RCM, 37°C, anaerobic	−
*Bifidobacterium pseudocatenulatum* MB0041		MRS/RCM, 37°C, anaerobic	−
*Bifidobacterium pseudocatenulatum* NCIMB 8811		MRS/RCM, 37°C, anaerobic	−
*Bifidobacterium* subsp. *infantis* MM0353		MRS/RCM, 37°C, anaerobic	−

*Inhibition is represented as zones of inhibition **+** = 0.5–2 mm halo, **++** = 2–4 mm halo, **+++** = > 4 mm halo and – = no inhibition.

The minimal inhibitory concentration (mic) of purified pseudocin 196 against *L. cremoris* HP, *S*. *dysgalactiae* GpB, *S*. *agalactiae* ATCC 13,813, *C. lituseburense* ATCC 25,759, and *C. perfringens* ATCC 362 was determined. *L. cremoris* HP was the most sensitive strain tested, correlating with the results obtained in the well diffusion assays, while *C. perfringens* ATCC 3626 was the least sensitive (Table 2). No comparative result was obtained for *C. perfringens* as the production of gas as a result of their fermentation prevented a reliable result when inoculated in agar. The values represented correspond to the average of two biological independent experiments.

**Table 2.** Minimal inhibitory concentration assessed in triplicate in a 96-well plate.

Strain	µM	µg/ml
*Lactococcus cremoris* HP	0.2	0.52
*Streptococcus dysgalactiae* GpB	0.78	2.09
*Streptococcus agalactiae* ATCC 13,813	3.13	8.37
*Clostridium lituseburense* ATCC 25,759	6.25	16.74
*Clostridium perfringens* ATCC 3626	12.5	33.48

### Functional analysis of the psc gene cluster

**Figure 5.**
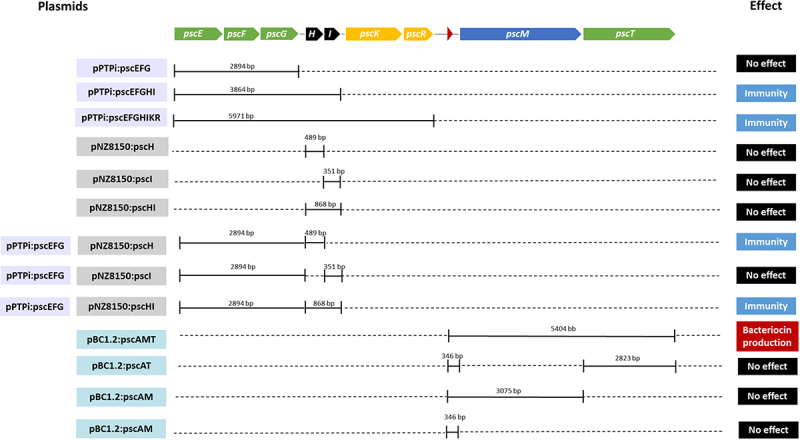
Schematic representation of DNA fragments cloned into the different vectors. Solid lines represent the regions that were cloned in a particular cloning vector, being pPtpi and/or pNZ8150 for expression in *L. cremoris* or pBC1.2 to allow expression in *B. breve* UCC2003. As indicated by colour coding on the left-hand margin, (purple) pPtpi constructs, (grey) pNZ8150 constructs and (blue) pBC1.2 constructs. Names were assigned based on the vector used and the genes included in each construction. The last column summarises the phenotypic effect as a result of heterologous expression of genes cloned into the corresponding host.

Lantibiotic gene clusters contain genes necessary for the production, modification, regulation and immunity of the bacteriocin they encode. Initial predictions indicate that the *psc* gene cluster of *B. pseudocatenulatum* MM0196 is divided into three functional regions. To confirm these predicted functions, particular gene sets of the cluster were cloned and assessed phenotypically. The genes believed to be involved in bacteriocin production, modification and transport were cloned into plasmid pBC1.2 to facilitate heterologous expression in *B. breve* UCC2003, while genes predicted to be responsible for conferring immunity were cloned into either pPTPi or pNZ8150 and were introduced alone or in combination to allow heterologous expression in *L. cremoris* NZ9000. An overview of the various constructed plasmids and their encoded properties is shown in Figure 5.

To validate the predicted function of *pscA* (MM0196_0190), *pscM* (MM0196_0191), and *pscT* (MM0196_0192), these genes were cloned into a low copy number shuttle vector able to replicate in *Escherichia coli* and *Bifidobacterium*, while also including a constitutive promoter.^42^ Derivatives of plasmid pBC1.2 containing various gene combinations were transformed into *B. breve* UCC2003, which is insensitive to pseudocin 196 and does not produce antimicrobial activity against the tested indicators.

**Figure 6.**
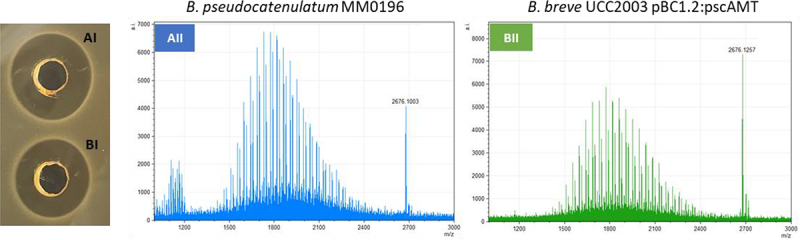
Well diffusion assay. C18 SPE cell free supernatants from *B. pseudocatenulatum* MM0196 (aI) and *B. breve* UCC2003 pBc1.2:laAMT (bI). MALDI-TOF MS spectra of C18 SPE extracts of cell free supernatant confirm the presence of the pseudocin 196-corresponding molecular mass in both *B. pseudocatenulatum* MM0196 WT (aII) and *B. breve* UCC2003 pBc1.2:laAMT (bII).

*B. breve* UC2003 recombinant derivatives containing either plasmid pBC1.2 (negative control), pBC1.2:pscAMT, pBC1.2:pscAT, pBC1.2:pscAM, or pBC1.2:pscA were constructed, and their respective CFS were tested by well diffusion assay for antimicrobial activity against the indicator, *L. cremoris* HP. Only *B. breve* UCC2003 harboring plasmid pBC1.2:pscAMT was able to produce a zone of inhibition (Figure 6(bI)) comparable to the WT strain (Figure 6(aI)) with no clearing zones observed for pBC1.2:pscAT, pBC1.2:pscAM, or pBC1.2:pscA indicating that all three *pscAMT* genes are required for pseudocin 196 production. MALDI TOF MS analysis of C18 SPE extracts of CFS from *B. pseudocatenulatum* MM0196 (Figure 6aII) and *Bifidobacterium breve* UCC2003 pBC1.2:pscAMT (Figure 6bII) both show the presence of the pseudocin 196-associated molecular mass, thereby confirming production of this bacteriocin by the recombinant strain. Pseudocin 196 was then purified from equal volumes of CFS from both *B. pseudocatenulatum* MM0196 and *B. breve* UC2003 pBC1.2:pscAMT and MALDI-TOF MS analysis of the C18 SPE showed that both contained an antimicrobial compound with a molecular mass of 2676 Da, thus corresponding to the molecular mass of pseudocin 196. Pseudocin 196 was purified to homogeneity by reversed phase HPLC and the active HPLC fractions were shown to contain antimicrobial activity consistent with the pseudocin 196 mass (data not shown), thereby confirming that the gene products of *pscA*, *PscM*, and *pscT* are all required for the production of the biologically functional lantibiotic.

**Figure 7.**
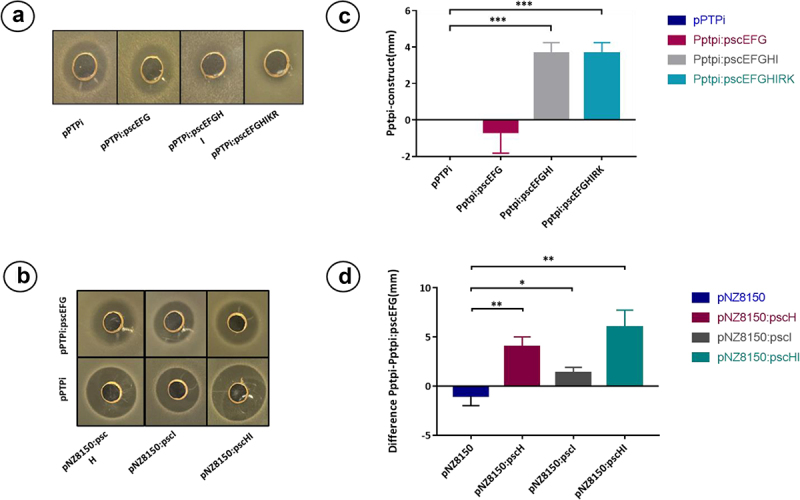
Well diffusion assay of constructed derivatives of *L. cremoris* NZ9000. Panels A and B well diffusion assay using the strains containing the constructs as indicator strains. Panels C and D show the difference of inhibition zone in mm in the different cases, the data corresponds to the average of three independent experiments. *p < .05, **p < .01, ***p < .001 by the pair t-test with the control group in each case.

To investigate which genes of the *psc* gene cluster contribute to immunity, different constructs, i.e., *psc*EFG, *psc*EFGHI, and *psc*EFGHIKR, containing the predicted immunity genes *pscE, pscF*, and *pscG*, were cloned into plasmid pPTPi, which contains a nisin-inducible promoter. The resulting pPTPi derivatives were then introduced into *L. cremoris* NZ9000 as this strain is sensitive to pseudocin 196 in well diffusion assays. The resulting recombinant strains were used as indicators in well diffusion assays to assess their sensitivity to CFS containing pseudocin 196. The assays showed that pPTPi:pscEFGHI and pPTPi:pscEFGHIKR displayed a smaller zone of inhibition when expression of the genes was induced with 10 ng/ml of nisin, while pPTPi:pscEFG did not show any difference compared with the inhibition zone for the strain containing the empty pPTPi plasmid (Figure 7, Panels A and C). These results suggest that *pscH* and *pscI* play a key role in providing immunity to pseudocin 196. To further investigate their role, both genes were cloned individually and together into a different vector, pNZ8150, also under a nisin-inducible promoter, and they were subsequently transformed into *L. cremoris* NZ9000 containing either pPTPi empty or pPTPi:pscEFG. The results of the well diffusion assay using these recombinants are shown in Figure 7(bB,dD) and demonstrate that the strain containing pNZ8150:pscH and pPTPi:pscEFG exhibits reduced zones of inhibition when compared with the strain containing pPTPi and pNZ8150:H, suggesting they confer a degree of immunity on the strain, while the strain containing pNZ8150:pscI either with pPTPi or pPTPi:pscEFG did not show any significant difference in inhibition zone size. To confirm that this combination of genes in both plasmids had the previously described effect, the strain containing pNZ8150:pscHI and pPTPi:pscEFG was tested by well diffusion assay, where a reduction of the inhibition zone was observed, similar to what was previously described in the strain harboring the construct pPTPi:pscEFGHI. No reduction of pseudocin 196 sensitivity was observed when the strains containing pNZ8150:pscH, pNZ8150:pscI, and pNZ8150:pscHI constructs were tested individually. Despite not being able to observe complete immunity against the peptide, possibly due to the fact that the *psc* genes, which originate from a high GC *Bifidobacterium* host, may not express well in the low GC lactococcal host, we conclude that *pscE*, *pscF*, *pscG*, and *pscH* are involved in pseudocin 196 immunity of the producer strain and a combination of the four genes is needed for optimum function. Summaries of the constructs tested can be found in Supplementary Table S2.

## Discussion

Bacteriocins have the potential to combat the rising problem of antibiotic resistance, which, according to the World Health Organisation, is one of the biggest threats to global health. Their specificity along with their robustness makes bacteriocins an attractive alternative to traditional treatments, not only as therapy, but also as disease prevention, especially when the producers are human gut commensals and may elicit other health benefits. Members of the genus *Bifidobacterium* have been studied extensively due to their purported beneficial effects for their human host, especially at early life, where a bifidobacteria-rich microbiota is correlated with a healthy infant gut, and its positive association with preventing long-term health problems, such asthma and allergies. Their high abundance and prevalence in the neonatal gut is facilitated by their ability to degrade complex dietary carbohydrates, which their host is unable to process. In addition to their fermentative activity, which may benefit the host through pH reduction and short chain fatty acid production, bifidobacterial strains may elicit other characteristics such as the production of vitamins, phytases and isoflavone-glycosides.^43,44^

To date, only a small number of bifidobacterial strains have been reported to produce an active antimicrobial peptide or bacteriocin despite the fact that main strains carry gene clusters putatively involved in this function in their genome. Pseudocin 196 is the first bacteriocin described for the species *B. pseudocatenulatum*, which is an unusual trait not only for this species but for the whole genus, as only eight potential antimicrobial peptides have been described to date none of which were studied in any particular detail.^13^ The reason behind this rarity of bacteriocin production by *Bifidobacterium* spp. is unknown but highlights the important and rather unique findings herein described.

The absence of bacteriocin production in a strain containing the gene cluster is not unique to members of the genus *Bifidobacterium*, as it has also been commonly observed for other Gram-positive bacteria such as *Lactobacillus*.^45^ There could be many reasons why the presence of a cluster does not correspond with the production of an active peptide, for example, the regulation of the operon might need activators from the environment that are lacking in our *in vitro* assay, or the presence of mutations in a non-coding region may affect regulation of bacteriocin production, or the operon may simply be switched off. Alternatively, the bacterial strain used as a screening indicator may not be sensitive to the bacteriocin, causing such antimicrobial being overlooked.

The rarity of bacteriocin-producing gene clusters in *B. pseudocatenulatum* suggests that this trait was recently acquired by strain MM0196 and that the *psc* gene cluster may have originated from other Gram-positive bacteria within the human microbiome. The presence of transposases flanking the cluster and the lower GC content of this genomic region when compared with the average of the genome are consistent with the occurrence of horizontal transfer. Other lantibiotic gene clusters including lacticin 481 (encoded between transposases) are also located on mobile elements.^40^

Lantibiotic type II gene clusters such as those associated with the production of lacticin 481 and nukacin ISK-1, are structurally similar to that of pseudocin 196 in that they both harbor a pro-peptide-encoding *lanA* followed by *lanM* and *lanT* genes and three putative immunity genes, *lanE, lanF* and *lanG*.^41,46^ Interestingly, the gene order in the pseudocin 196 gene cluster is different, while four additional genes, *pscH, pscI, pscK*, and *pscR*, are present between *lanE, lanF* and *lanG*, and the *lanA, lanM* and *lanT* genes.

**Figure 8.**
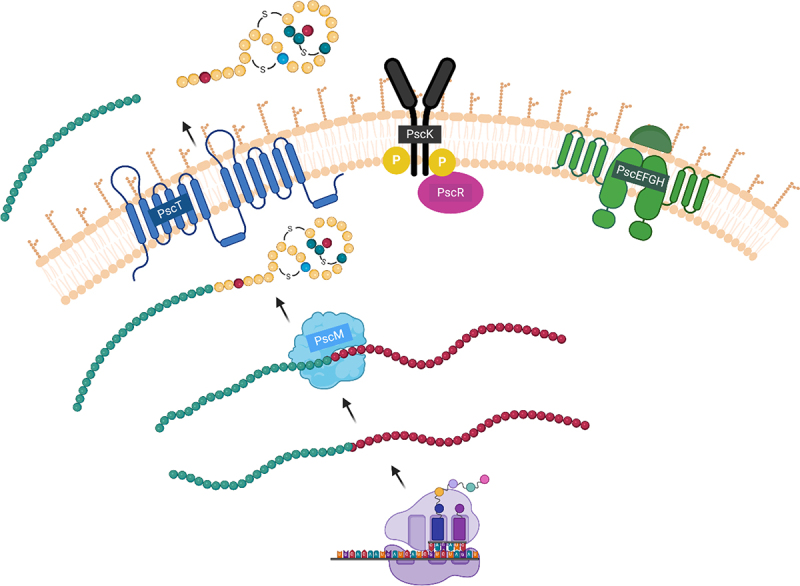
Proposed model for the biosynthesis of pseudocin 196. Pro-peptide is modified by PscM and transported outside the cell by PscT as an active peptide. PscK and PscR, are potentially regulating the expression and production of the peptide. The transport system PscEFGH which is involved in the immunity is potentially excreting pseudocin 196 externally.

Based on our own findings and those reported for other lantibiotics, we propose that pseudocin 196 is initially produced as a pro-peptide and modified by PscM, an enzyme responsible for introducing chemical alterations in amino acids involved in structural ring formation. The protease domain of PscT then cleaves off the signal peptide and exports the mature peptide to the outside of the cell. We show furthermore that immunity to pseudocin 196 is provided by the products of four genes: *PscE, pscF, PscG*, and *pscH*, though the precise immunity mechanism was not investigated in this study (Figure 8). However, immunity genes have been reported to encode an ABC transporter system that expels the lantibiotic from the membrane, thereby avoiding membrane disruption. For example, in the case of nisin, four genes are involved in immunity; *nisFEG* and *nisI*, where *nisFEG* encode an ABC transporter and *nisI* encodes an immunity protein that binds the peptide, and either of these can provide immunity to nisin independently.^41^ However, in our study we observed that *pscEFG* and *pscH* cannot provide immunity independently, which makes us conclude that the mechanism will differ from that previously described in nisin. Potentially, PscH needs to bind the peptide in order to present this then to the PscEFG transport system to remove it from the cytoplasmic membrane to avoid membrane disruption. However this notion will require further experimental exploration.

Lantibioic production may be under the regulatory control of a bacteriocin-specific two-component histidine kinase response regulator system (2CS) encoded by *lanRK*.^47^ In the case of the pseudocin 196 gene cluster, a 2CS is predicted to be encoded by *pscK* and *pscR*, suggesting that the pseudocin 196 peptide regulates its own expression similar to nisin expression in *Lactococcus*. A 2CS-mediated, lantibiotic-inducible expression system may also be constructed to be employed in *B. pseudocatenulatum* based on elements of the pseudocin 196 cluster, similar to that of *L. lactis* and nisin.^48,49^

The ability of pseudocin 196 to inhibit microorganisms such as *C. perfringens* involved in NEC pathogenicity and *S. agalactiae*, which can cause vaginal infection in pregnant women and which may be transmitted to the new-born through the birth canal and cause neonatal death, suggest clinical potential.^28,32^ The prophylactic use of *B. pseudocatenulatum* MM0196 in pregnant women may not only be able to prevent this infection from being transmitted to the offspring but could also have a positive impact on the mother and/or the baby, derived from the benefits associated with a *Bifidobacterium* rich gut microbiota.

Preclinical studies are required to verify the potential of this strain to colonize the gut when administered externally and to determine the effect that the strain might have in positively influencing the microbial composition of this niche. Toxicity assays are required to determine its safety, however very positive results have been observed in previous studies on similar peptides where the absence of cell death was observed after exposing epithelial cells to the antimicrobial peptide.^50,51^

In summary, by a combination of comparative genome analysis and purification techniques, we identified, and characterized a novel lantibiotic encoded by a gene cluster encompassed by a *B. pseudocatenulatum* strain. Additionally, by heterologous gene expression, we have identified the genes responsible for producing a biologically active peptide as well as those needed to confer immunity to the peptide, thus representing the most complete study of a bacteriocin produced by *Bifidobacterium* spp. to date.

## Materials and methods

### *Isolation and culture conditions of* B. pseudocatenulatum *MM0196*

*B. pseudocatenulatum* MM0196 was isolated from the feces of a healthy pregnant woman at week 32 gestation. The subject was enrolled in the MicrobeMom study, aimed at identifying mother-infant bifidobacterial strain transmission (trial reference number: ISRCTN53023014). Sample collection took place in the Maternity Hospital in Dublin, and the sample was stored at −20°C until processing. 100 mg of the frozen fecal sample was resuspended in 1 ml of PBS (Sigma Aldrich, Ireland) supplemented with 0.05% L-cysteine hydrochloride (Sigma Aldrich, Ireland). The fecal resuspension was plated on 10 plates of Man Rogosa and Sharpe (mMRS) agar (Watson et al. 2013) prepared from first principles and supplemented with 0.5% (wt/vol) lactose (Sigma Aldrich, Ireland), 0.05% L-cysteine-HCl (Sigma Aldrich, Ireland), 100 μg/ml mupirocin (Carbosynth, Ireland), 1 mM ferulic Acid (Sigma Aldrich, Ireland), 5 μg/ml hemin (Sigma Aldrich, Ireland), and 50 μg/ml nystatin suspension (Sigma Aldrich, Ireland). Agar plates were incubated under anaerobic conditions in a modular atmosphere-controlled system (Davidson and Hardy, Belfast, Ireland) at 37°C for 48–72 h. Colonies were resuspended in 1 ml of PBS supplemented with 0.05% L-cysteine-HCl, and serial- diluted in PBS using a 96-well plate, dilution between 1 × 10e-4 and 1 × 10e-9 were spread on mMRS agar supplemented as previously described. After 48 h incubation as described above, individual colonies were selected and re-streaked twice, using the T-streak method to obtain individual colonies using the same agar-medium following 48 h anaerobic incubation. mMRS broth media supplemented with 0.5% lactose and 0.06% cysteine-HCl, was inoculated with an individual colony. Pure culture was stocked in 0.2% (vol/vol) glycerol (Fisher Bioreagent, Ireland) and stored at −80°C. The isolate was confirmed using the fructose-6-phosphate phosphoketolase activity test F6PPK activity (Scardovi, V. *Genus Bifidobacterium*. 1418–1434.^52^

### Bacterial strains and culture conditions

*B. pseudocatenulatum* MM00196 was routinely cultured under anaerobic conditions at 37°C in modified de Man Rogosa Sharpe (mMRS) prepared from first principles and supplemented with 0.5% lactose and 0.06% cysteine-HCl (Watson et al. 2013). Indicator strains, their culture media, and growth conditions are listed in Table 1. The media used are as follows: Man Rogosa Sharpe (MRS) from Difco, BD, Le Pont de Claix, France), Reinforced Clostridium Medium (RCM)(Oxoid, Ltd., Basingstoke, England) Brain Heart Infusion (BHI) (Oxoid Ltd., Basingstoke, England) and M17 (Ltd., Basingstoke, England), supplemented with 0.5% (wt/vol) glucose (Sigma Aldrich, Ireland) referred below as GM17.

Agar (Neogen, UK) was added (1.5% w/v) to the media specific to the strain when agar plates were required and 1.5% and at 0.7% w/v for well diffusion assays to facilitate diffusion of the suspension.

### Genome sequencing and annotation

DNA extraction for Illumina sequencing was performed from 1.5 ml of an overnight culture using the Genelute bacterial genomic DNA kit (Sigma). Complete genome sequencing of the isolate MM0196 was achieved using a combination of an Illumina MiSeq platform (GenProbio) and a Pacific Bioscience Pacbio Sequel platform (PacBio, Oslo, Norway). The obtained raw reads were assembled with the Hierarchical Genome Assembly Process (HGAP) pipeline using the protocol RS_Assembly.2 implemented in SMRT Smart Analysis portal v.2.3 (https://www.pacb.com/support/software-downloads/, accessed on 4 May 2021).^53^ In order to achieve a complete circular genome, paired reads from the Illumina platform and filtered subreads from the Pacbio platform were assembled using Unicycler v0.4.8-beta in bold mode. Open Reading Frame (ORF) prediction and automatic annotation was performed using Prodigal v2.0 (http://prodigal.ornl.gov) for gene predictions, BLASTP v2.2.26 (cutoff e-value of 0.0001)71 for sequence alignments against a combined bifidobacterial genome-based database, and MySQL relational database to assign annotations. Predicted functional assignments were manually revised and edited using similarity searches against the non-redundant protein database curated by the National Centre for Biotechnology Information (ftp://ftp.ncbi.nih.gov/BLAST/db/) and PFAM database (http://pfam.sanger.ac.uk.), allowing a more detailed, *in silico* characterization of hypothetical proteins. GenBank editing and manual inspection was performed using Artemis v18 (http://www.sanger.ac.uk/resources/soft-ware/artemis/). Transfer RNA genes were identified employing tRNAscan-SE v1.4 and ribosomal RNA genes were detected based on Rnammer v1.272 software supported by BLASTN v2.2.26.

### Bacteriocin cluster identification and phylogeny

The bacteriocin and RiPPs mining tool BAGEL 4 was used to identify the putative bacteriocin cluster confirmed by manual analysis of bacterial genomes using the visualization tool ARTEMIS.^35,54^ The phylogenetic tree was built based on a multiple sequence analysis in clustalw, using bootstrap values based on 1000 replications and the resulting nexus tree file was input into the visualization tool iTOL to generate the figure where tree was rooted to the most distant sequence of the set (NQ170421.1).^55^ The tree includes the first hundred sequences as a result of a non-redundant blastp using the amino acid sequence of the bacteriocin as input.

### Cloning and heterologous expression of the operon

For the construction of recombinant plasmids, various DNA fragments containing the targeted genes were amplified with Q5 High Fidelity polymerase (New England Biolab, UK) using MM0196 chromosomal DNA extracted with Genelute bacterial genomic DNA kit (Sigma) as a template. Using the primers specified in Table 3. The amplicon pscAMT and plasmid pBC1.2 were digested with HindIII and Xbal (New England Biolabs, UK). pscEFG, pscEFGHI, pscEFGHIKR and plasmid pPTPi were digested with BamHI and MluI (New England Biolabs, UK). And the amplicons pscH, pscI, pscHI and plasmid pNZ8150 were digested with HindIII-HF and Xbal (New England Biolabs, UK). All PCR products and their corresponding plasmids were ligated using T4 ligase (New England Biolabs, UK). The ligation mix was introduced by electroporation into *E. coli* EC101 and transformants were selected with 10 ug/ml chloramphenicol in pBC1.2 and pNZ8150 constructs while 10 ug/ml of tetracycline was used to select pPTPi constructs.

**Table 3.** Primers used in this study.

Primer name	Sequence (5’-3’)	Description
**0190XbaI-Fw**	TGCATC**TCTAGA***TTGACAGCTAGCTCAGTCCTAGGTATAATGCTAGC*GAAAAGACCGTGACAACGG	To amplify fragment containing *pscAMT*
**0192Pst1-Rv**	TGCCTC**CTGCAG**GACGGTGTTCAGATTAAGTG	To amplify fragment containing *pscAMT*
**183 BamHI Fw**	TGCATC**GGATCC**AGGAGGCACTCACGACGATAATGACAATAC	To amplify fragment containing *pscEFG*, *pscEFGHI* and *pscEFGHIKR*
**185 MluI Rv**	TGCCTC**ACGCGT**CTTTCCATGGTGTTGGGTG	To amplify fragment containing *pscEFG*
**187 MluI Rv**	TGCCTC**ACGCGT**GATTCTCCTTCCCTGGGT	To amplify *pscEFGHI* fragment
**189 MluI Rv**	TGCCTC**ACGCGT**CACGTGAGAAAATACCGGTC	To amplify fragment containing *pscEFGHIKR*
**186 XbaI Fw**	TGCATC**TCTAGA**AGGAGGCACTCACGGCTTCATCATGCACA	To amplify fragment containing *pscH* and *pscHI*
**186 HindIII Rv**	TGCCTC**AAGCTT**GGTCATTGCTCCTTGGAT	To amplify fragment containing *pscH*
**187 XbaI Fw**	TGCATC**TCTAGA**AGGAGGCACTCACCCATGGCAAACACATC	To amplify fragment containing *pscI*
**187 HindIII Rv**	TGCCTC**AAGCTT**CGTTGTCAGTCGGATT	To amplify fragment containing *pscI* and *pscHI*
**Intern 190–191 Fw**	GACAACGGTGGCTCCTCCTGC	Confirmation of recombinant in *B. breve* UCC2003
**Intern 190–191 Rv**	CCAGACAAGCTTCCTCTGAC	Confirmation of recombinant in *B. breve* UCC2003
**pPTPi_Fw**	TGATTTCGTTCGAAGGAACTA	Multicloning site pPTPi
**pPTpi_Rv**	TGGCGGACAATAAGTCCTC	Multicloning site pPTPi

Restriction sites incorporated into oligonucleotide primer sequences are indicated in bold and promoter regions incorporated into nucleotide primer sequences are indicated in italics.

In the case of pPTPi:pscEFG, pPTPi:pscEFGHI, pPTPi:pscEFGHIKR, pNZ8150:pscH, pNZ8150:pscI and pNZ8150:pscHIand constructs, 20 ng of plasmid DNA was transformed by electroporation into *L. cremoris* NZ9000. The plasmid content of the transformants was verified by PCR and the different clones were selected for phenotypic analysis. All the strains used in this study can be found in the Table 4.

**Table 4.** Plasmids and strains used for cloning in this study.

Strains	Description	Reference
*Escherichia coli* EC101	Cloning host	^44^
*Bifidobacterium breve* UCC2203	Cloning host	^45^
*Lactococcus cremoris* NZ9000	Cloning host	^46^
**Plasmids**	**Description**	**Reference or source**
pBC1.2	Cm^r^; cloning vector	Laboratory collection
pBC1.2:pscAMT	Cm^r^; containing *pscA*, *pscM* and *pscT* genes	This study
pBC1.2:pscAT	Cm^r^; containing *pscA* and *pscT* genes	This study
pBC1.2:pscA	Cm^r^; containing *pscA* gene	This study
pBC1.2:pscMT	Cm^r^; containing *pscM* and *pscT* genes	This study
pPTPi	Tet^r^, cloning vector carrying nisin inducible promoter	This study
pPTPi:pscEFG	Tet^r^, containing *pscE*, *pscF* and *pscG* genes	This study
pPTPi:pscEFGHI	Tet^r^, containing *pscE*, *pscF, pscG, pscH* and *pscI* genes	This study
pPTPi:pscEFGHIKR	Tet^r^, containing *pscE*, *pscF, pscG, pscH, pscI, pscK* and *pscR* genes	This study
pNZ8150	Cm^r^, cloning vector carrying nisin inducible promoter	Commercially available
pNZ8150:pscH	Tet^r^, containing *pscH* gene	This study
pNZ8150:pscI	Tet^r^, containing *pscI* gene	This study
pNZ8150:pscHI	Tet^r^, containing *pscH* and *pscI* gene	This study

### Antimicrobial activity assays

#### Well diffusion assay

The cell-free supernatant obtained from an overnight culture of the strain tested was used for the well diffusion assays. Cell free supernatants were obtained by centrifugation of 10 ml from an overnight culture for 5 minutes at 4°C and 55,000 rpm and filtering through a 0.2 µm pore size filter. For the antimicrobial assay, 20 ml of 0.7% agar of appropriate media inoculated with 200 µl of an overnight culture from the indicator strain were poured in a petri dish. A sterile tip was used to make wells in the agar plate prior addition of 50 µl volume of pH neutralized supernatant using 5 M NaOH. Plates were incubated at room temperature in the laminar flow hood until the supernatant diffused into the agar, followed by overnight incubation according to the optimum condition for the indicator strain (Table 1). After 24 hours the zones of inhibition were observed and measured. Assays were performed in triplicate.

#### Overlay assay

Ten microliters of an overnight culture was spotted into an agar plate of RCA and grown anaerobically for 48 hours at 37 degrees. The culture was exposed to UV light for 15 minutes, in order to kill the bacterial cells. An overlay of 4 ml 0.7% agar inoculated with 200 µl of an overnight culture from the indicator strain, was poured into the plate and incubated according to the indicator optimal growth conditions. After 24 hours a halo of inhibition can be observed around the bacterial spot if the organism was producing an antimicrobial compound.

### *Colony mass spectrometry of* B. pseudocatenulatum *MM0196*

Isolated colonies from an agar plate were mixed with 50 µl 70% propan-2-ol 0.1% TFA, vortexed three times and centrifuged at 14,000 rpm x 30 seconds. MALDI TOF Mass spectrometry was performed on the cell extract using a Bruker Autoflex MALDI TOF mass spectrometer (Bruker, Bremen, Germany). A 0.5-µl aliquot of matrix solution (α - cyano 4-hydroxy cinnamic acid, 10 mg/ml in acetonitrile-0.1% (v/v) trifluoroacetic acid) was deposited onto a stainless steel target and left for 10 seconds before being removed. The residual solution was allowed to air-dry and 0.5 µl sample solution was deposited onto the pre-coated sample spot. 0.5 µl of matrix solution was added to the deposited sample and allowed air-dry. The sample was subsequently analyzed in positive-ion reflectron mode.

### Purification of pseudocin 196 from cell free supernatant for confirmation of bacteriocin production by *B.* *breve* UCC2003 containing pBc1.2:pscAMT

Three milliliters of cell free supernatant from an overnight culture of *B. pseudocatenulatum* MM0196 was applied to a 200 mg Strata – E C18 SPE column (Phenomenex, Cheshire, UK) pre-equilibrated with methanol and water. The column was washed with 3 ml of 30% ethanol and antimicrobial activity eluted with 2 ml 70% 2-propanol 0.1 TFA. The 70% 2-propanol 0.1 TFA eluent was assessed for the pseudocin 196 mass by MALDI TOF mass spectrometry as described above.

### Purification of pseudocin 196 from cell free supernatant for assays

Two liters of cell free supernatant was applied to an Econo column (Bio-Rad Laboratories, California, USA) containing 60 g Amberlite XAD16N (Sigma-Aldrich, Missouri, USA). The column was washed with 450 ml of 40% ethanol and antimicrobial activity eluted with 450 ml of 70% propan-2-ol 0.1% TFA. The propan-2-ol was removed from the XAD IPA eluent by rotary evaporation (Buchi AG, Flawil, Switzerland) and the resulting sample applied to a 10 g Strata C18-E SPE column pre-equilibrated with methanol and water. The column was washed with 90 ml 35% ethanol and antimicrobial activity eluted in 60 ml 70% propan-2-ol 0.1% TFA. The propan-2-ol was removed from the C18 SPE 70% propan-2-ol 0.1% TFA eluent and the sample applied to a semi preparative Jupiter Proteo RP-HPLC column (250 × 10 mm, 4µ, 90Å, Phenomenex, Cheshire, UK) running a 30–55% acetonitrile 0.1% TFA gradient at 2.5 ml/min over 45 minutes where mobile phase A is 0.1% TFA and mobile phase B is 90% acetonitrile 0.1% TFA. Eluent was monitored at 214 nm and fractions collected at 1 minute intervals. Fractions were assayed for antimicrobial activity and active fractions checked for the pseudocin 196 mass using MALDI TOF Mass Spectrometry. Fractions deemed purest were pooled and lyophilized.

### Minimum inhibitory concentration

The MIC of the purified pseudocin 196 was determined in triplicate in a 96-well plate (Sarstedt Ltd, Drinagh, Ireland). Purified Pseudocin was resuspended in sterile water to a final concentration of 100 µM and two fold serially diluted to a concentration of 0.98 µM. Fresh media was inoculated with an overnight culture of the indicator strain until OD_600 nm_ of ~0.5 was reached: 100 µl of media with of 10^5^ CFU/ml was transferred to the well plate containing the different dilutions of the bacteriocin. The plate was incubated overnight, and MIC determined as the lowest concentration that inhibited the growth of the indicator strain. Two independent biological replicates were performed.

### Nucleotide sequence and accession number

The full genome sequence of *B. pseudocatenulatum* MM0196 was deposited at the GenBank under Accession No. JAWNGD000000000.

## Authors contribution

RSG carried out all the experimental work except for the peptide identification by MALDI-TOF and the purification of the peptides that were performed by POC. ION performed the BAGEL prediction. Manuscript was prepared by RSG, POC and DVS. PC, BM, CL participated in the design and guidance of the study. RM and FM provided the samples where the producer strain was isolated from. All authors read and approved the final manuscript.

## Supplementary Material

Supplemental Material

## Data Availability

The authors confirm that the data supporting the findings of this study are available within the article and its supplementary materials.
