# Correction

**DOI:** 10.1080/19490976.2024.2409028

**Published:** 2024-10-03

**Article title**: Pseudocin 196, a novel lantibiotic produced by Bifidobacterium pseudocatenulatum elicits antimicrobial activity against clinically relevant pathogens

**Authors**: Sanchez-Gallardo, R., O’Connor, P. M., O’Neill, I. J., McDonnell, B., Lee, C., Moore, R. L., McAuliffe, F.M., Cotter, P.D., and van Sinderen, D.

**Journal**: *KGMI: Gut Microbes*

**DOI**: https://doi.org/10.1080/19490976.2024.2387139

The article was originally published without the reference 18 (Javvadi SG, et al 2022).

The new reference below has been included in the original article, and the remaining references have been renumbered. The article has been republished accordingly.

18. Javvadi SG, Kujawska M, Papp D, Gontarczyk AM, Jordan A, Lawson MAE, et al. A novel bacteriocin produced by Bifidobacterium longum subsp. infantis has dual antimicrobial and immunomodulatory activity [Internet]. Microbiology; 2022 Jan [cited 2023 Nov 25]. Available from: http://biorxiv.org/lookup/doi/10.1101/2022.01.27.477972

