# Supplementary material for: Pseudocin 196, a novel lantibiotic produced by Bifidobacterium pseudocatenulatum elicits antimicrobial activity against clinically relevant pathogens
Source: Gut Microbes. 2024 Aug 6;16(1):2387139. doi: 10.1080/19490976.2024.2387139 (PMC11305057; doi:10.1080/19490976.2024.2387139)
Supplement: Supplemental Material [file KGMI_A_2387139_SM9772.zip › Supplementary material.docx]

Supplementary material

**Table 1.** In silico analysis of the predicted function of each gene present in the cluster.

| **Gene ID** | **Gene name** | **Gene size (bp)** | **Size (kDa)** | **TM** | **Homology** | **Putative function** |
| --- | --- | --- | --- | --- | --- | --- |
| MM0196_0183 | *pscE* | 1095 | 364 | 0 | ABC transporter | Immunity |
| MM0196_0184 | *pscF* | 840 | 279 | 6 | ABC transporter | Immunity |
| MM0196_0185 | *pscG* | 864 | 287 | 6 | ABC transporter | Immunity |
| MM0196_0186 | *pscH* | 489 | 162 | 1 | prokaryotic membrane lipoprotein  lipid attachment site | Unknown |
| MM0196_0187 | *pscI* | 351 | 116 | 2 | protein unknown function | Unknown |
| MM0196_0188 | *pscK* | 1290 | 429 | 5 | two-component system histidine kinase | Regulation |
| MM0196_0189 | *pscR* | 684 | 227 | 0 | two-component response regulator | Regulation |
| MM0196_0190 | *pscA* | 150 | 49 | 0 | Pre-peptide | Pseudocin 196 precursor |
| MM0196_0191 | *pscM* | 2700 | 899 | 0 | Lanthionine synthetase | Maduration |
| MM0196_0192 | *pscT* | 2076 | 691 | 5 | Protease ATP-dependent transporter | Secretion/Maduration |

**Table 2.** Summary of all the recombinant strains tested by well diffusion assay phenotypic effect observed. All *Lactococcus cremoris* NZ9000 strains were tested as indicators against *B. pseudocatenulatum* MM0196 supernatant. And *B. breve* UCC2003 supernatant was tested against *L. cremoris* HP

|  | **Plasmid/Construct** | **Effect** |
| --- | --- | --- |
| ***Lactococcus cremoris* NZ9000** | **No plasmid**  **pNZ8150**  **pNZ8150:lanH**  **pNZ8150:lanI**  **pNZ8150:lanHI**  **pPTPi**  **pPTPi:lan EFG**  **pPTPi:lanEFGHI**  **pPTPi:lanEFGHIKR** | None  None  None  None  None  None  None  Sensitivity reduction  Sensitivity reduction |
|  |  |  |
| ***Lactococcus cremoris* NZ9000 pPTpi** | **pNZ8150**  **pNZ8150:lanH**  **pNZ8150:lanI**  **pNZ8150:lanHI** | None  None  None  None |
|  |  |  |
| ***Lactococcus cremoris* NZ9000 pPTpi:lanEFG** | **pNZ8150**  **pNZ8150:lanH**  **pNZ8150:lanI**  **pNZ8150:lanHI** | None  Sensitivity reduction  None  Sensitivity reduction |
| ***Bifidobacterium breve* UCC2003** | **No plasmid**  **pBC1.2**  **pBC1.2:pscA**  **pBC1.2:pscAM**  **pBC1.2:pscAT**  **pBC1.2:ATM** | None  None  None  None  None  Bacteriocin production |

**
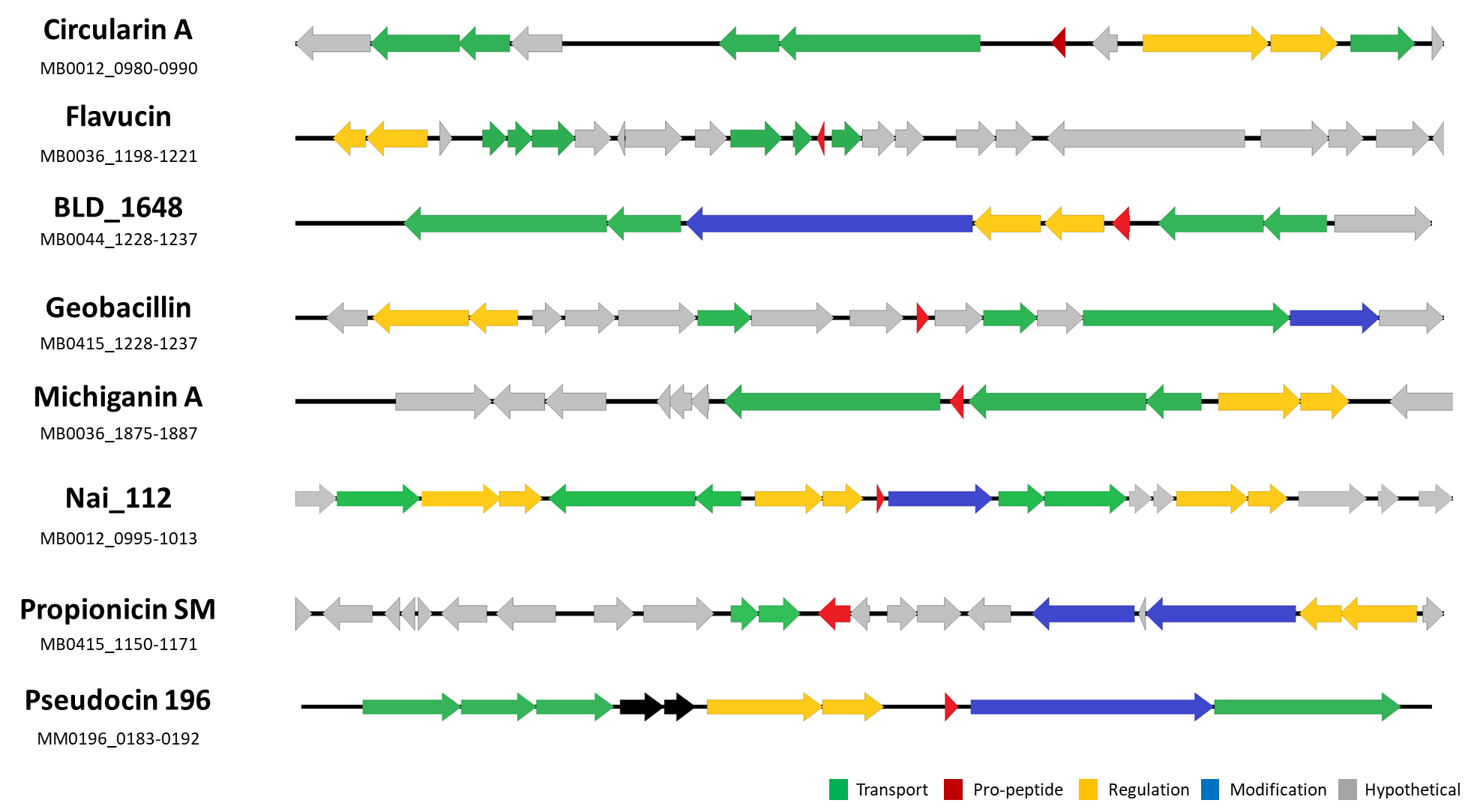
**


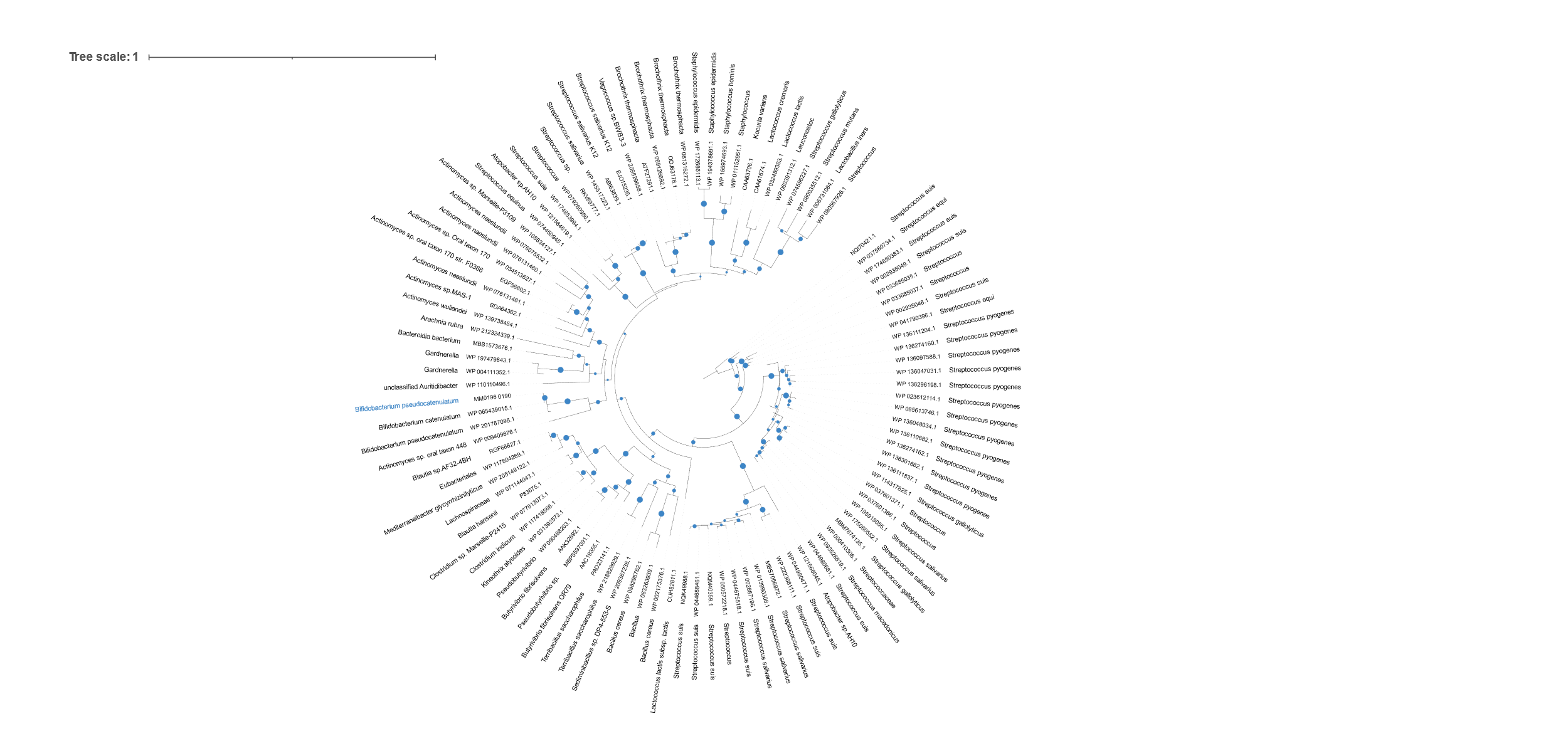
**Figure 1.** Schematic representation of the genomic structure of the bacteriocin clusters predicted in the different bifidobacterial genomes. A representative from each cluster was selected for this representation.

**Figure 2**. Phylogenetic tree of *spc*A structural genes based on the 100 results from a non-redundant blast using aminoacidic sequence. Size of the blue circles on the phylogenetic tree indicates bootstrap values.


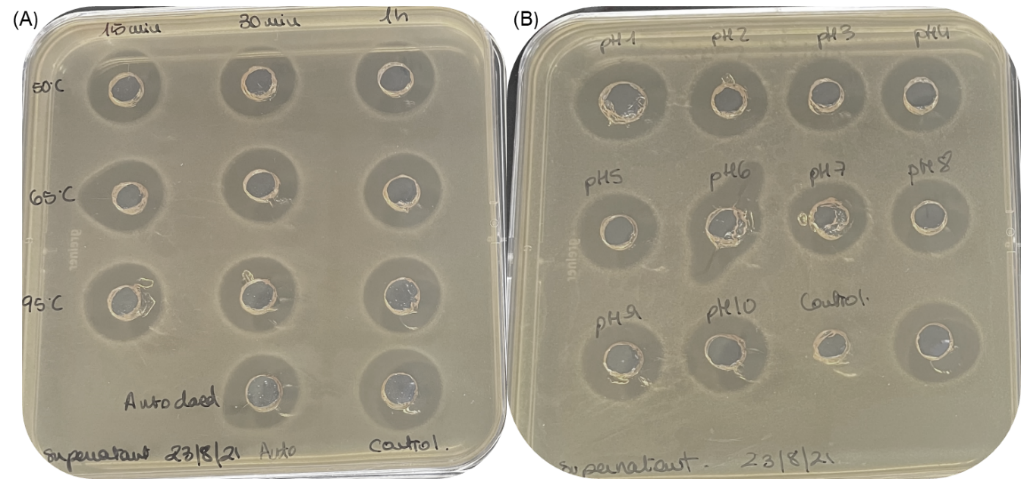


**Figure 3.** Stability of the peptide was determined by exposing the cell -free supernatant to different temperatures in various time intervals and pH. (A)Well diffusion assay of the supernatant at different exposed to different temperatures (50, 65, 95 degrees, as well as autoclaved) at different time intervals (15 min, 30 min and 60 min). (B) Well diffusion assay of cell free supernatant adjusted to a wide range of pH. Peptide is quite robust and is not susceptible to pH changes.
